# Global burden of Hodgkin lymphoma among children and adolescents: a population-based study using GBD 2021

**DOI:** 10.3389/fped.2025.1629229

**Published:** 2025-11-21

**Authors:** Jie Chen, Yue Wang, Jindou Guo, Yuqin Song, Jun Zhu, Jun Ma, Maigeng Zhou, Jinlei Qi, Weiping Liu

**Affiliations:** 1 Key Laboratory of Carcinogenesis and Translational Research (Ministry of Education), Department of Lymphoma, Peking University Cancer Hospital & Institute, Beijing, China; 2 National Center for Chronic and Noncommunicable Disease Control and Prevention, Chinese Center for Disease Control and Prevention, Beijing, China; 3 Department of Hematology and Oncology, Harbin Institute of Hematology and Oncology, Harbin, China

**Keywords:** Hodgkin lymphoma, global burden of disease, Age-Period-Cohort, socio-demographic index, children and adolescents

## Abstract

**Background:**

Hodgkin Lymphoma is a rare lymphatic system malignancy which has a negative impact on the health of children and adolescents. While high-income regions have seen a decline in mortality rates due to early diagnosis and advances in treatment strategies (e.g., chemotherapy, radiotherapy, and targeted therapy), low- and middle-income countries continue to face high mortality rates due to limited medical resources and delayed treatment.

**Methods:**

Employing data from the Global Burden of Disease (GBD) 2021 study, this analysis assessed the burden of Hodgkin lymphoma among children and adolescents from 1990 to 2021. Metrics including incidence, mortality, and disability-adjusted life years (DALYs) were evaluated across Socio-demographic Index (SDI) quintiles. Age-Period-Cohort (APC) and Nordpred models were used to analyze long-term trends and project future disease burden. Additionally, joinpoint regression analysis identified significant temporal shifts in trends throughout the study period.

**Results:**

The estimated DALY number was 265,016, and the ASDR among children and adolescents was 9.816 per 100,000 person in 2021. There were 8,700 new HL cases and 3,385 deaths, and the ASIR and ASMR were 0.320 and 0.125 per 100,000 population, respectively. The estimated number of prevalent HL cases was 50,128 worldwide with the ASPR of 2.001 per 100,000 population. Globally, the Hodgkin lymphoma burden was consistently higher in male children and adolescents than in females across all metrics. Results reveal a global decline in age-standardized incidence rates of HL among children and adolescents, with significant regional variations. Low- and middle-income regions experienced an increase in HL burden, while high-income regions showed a decline trend. The case number of HL in the children and adolescents increased from 8,283 in 1990 to 8,700 in 2021, while ASIR declined by 9.52%. The APC model projected a continued decline in HL incidence and mortality globally by 2040. However, health inequalities persist, with low-income countries bearing a disproportionately high burden of HL.

**Conclusions:**

The burden of HL among children and adolescents is increasing, with a significant difference by sex, age and regions, which highlight the urgent need for equitable diagnostic and therapeutic access in low-resource settings to meet the 2030 SDG targets on child health.

## Introduction

Hodgkin lymphoma (HL) exhibits distinct epidemiologic features with an age-specific incidence pattern that varies by socioeconomic status, with a peak in young adults (age 15–44 years) and a later peak in older adults (older than 55 years) in industrialized countries (1). HL was the third most common malignancy among adolescent aged 15–19 years and the seventh most common among children aged 0–14 years in the North American (2). HL exhibits striking geographical and socioeconomic disparities in disease burden (3). Globally, age-standardized mortality rates and disability-adjusted life years (DALYs) for HL have declined over recent decades. However, adolescent and young adults (15–39 years) in low-income regions continue to experience a disproportionate share of residual DALYs (1). A striking 8.3-fold mortality gap persists between high- and low-income countries, underscoring the urgent need for equitable access to diagnostics and treatment—even as novel therapies such as immune checkpoint inhibitors emerge (4–6).

HL in children differs meaningfully from adult-onset disease in terms of histology, EBV association, and clinical behavior. While prior research has predominantly focused on adult populations, it remains unclear in understanding HL burden among children and adolescents (7–9). It is important to emphasize that EBV-positive HL is more frequently observed in developing countries compared to developed Western nations, with particularly high incidence rates among younger children in resource-limited settings.

The Global Burden of Disease (GBD) 2021 database and Age-Period-Cohort modeling offer a robust framework for analyzing spatiotemporal disease dynamics and projecting future trends—crucial for targeting interventions in vulnerable youth populations.

This analytical approach provides a robust framework for elucidating disease dynamics and informing targeted strategies to reduce premature mortality from non-communicable diseases in this vulnerable population. By quantifying disparities in disease burden, this study provides empirical evidence to advance the global agenda of reducing inequalities and promoting health and well-being for all at all ages, notably through supporting efforts to reduce premature mortality from non-communicable diseases (SDG 3.4) and achieve universal health coverage (SDG 3.8).

## Methods

This study used GBD 2021 data and age-period-cohort modeling to analyze spatiotemporal trends in HL incidence, mortality, and DALYs specifically among children and adolescents. The GBD 2021 study delivers a rigorously standardized epidemiological accounting of 371 pathologies across 204 nations/territories.

All data cloud was accessible for free access through the GBD 2021 (https://ghdx.healthdata.org/gbd-2021/sources). This disability-weighted metric system quantifies health loss through triangulated incidence-prevalence-mortality modeling (10). All epidemiological datasets and computational codes are accessible via the Global Health Data Exchange (GHDx) repository, with comprehensive metadata documenting case definitions, modeling algorithms, and uncertainty analyses across GBD iterations (11). Sociodemographic Index (SDI) quintile stratification synthesizing per capita income, educational attainment, and fertility rates was employed to compare disease trajectories across development continua: low, low-middle, middle, middle-high, and high SDI cohorts (12).

### Age-Period-Cohort (APC) model analysis

To analyze long-term trends in the burden of HL and to project future trajectories, we employed an APC model. APC models are widely used in epidemiology to disentangle the effects of three temporal dimensions: age (reflecting physiological aging and life-course exposure), period (capturing external influences affecting all age groups simultaneously, such as diagnostic advances or treatment innovations), and cohort (representing the shared experiences of groups born in the same time interval, potentially reflecting early-life exposures or generational risks) (13). Model parameters were estimated using R version 4.2.2 with the “APC” package (v2.0.1).

The APC model estimated both the overall time trend and the incidence trend within each age group. The former is expressed as the annual percentage change in incidence, denoting the net drift (% per year), shaped by calendar time and continuous birth cohorts. The latter indicates the percentage change in annual incidence by age, termed as the local drift (% per year). Even minor shifts in drift values (% per year) can lead to significant alterations in the fitting rate over a 30-year period. Applied to pediatric and adolescent patients HL epidemiology, the model elucidates etiological period effects (e.g., therapeutic innovations post-2000) from cohort-derived susceptibilities (e.g., EBV exposure patterns), while quantifying age-specific incidence modulation across development phases.

### Study population

The study subjects were children and adolescents. The World Health Organization defined children and adolescents as people aged 20 and under. Additionally, adolescence was defined as the transitional period between childhood aged 10–19.

## Results

### Global burden of HL among children and adolescents in 2021

In 2021, the estimated DALY number and age-standard DALYs rate (ASDR) per 100,000 person-years were 265,016 and 9.816, respectively. The estimated number of children and adolescents prevalent HL cases was 50,128 worldwide. The age-standard prevalence rate (ASPR) was 2.001 per 100,000 population. There were 8,700 new cases and 3,385 deaths. The age-standard incidence rate (ASIR) and age-standard mortality rate (ASMR) were 0.320 and 0.125 per 100,000 population, respectively (Tables 1–4).

**Table 1:** Age-standardized incidences and average annual percentage change (AAPC) of Hodgkin lymphoma in pediatric and adolescents in 1990 and 2021.

Stratification	1990		2021		AAPC (95% CI)
	Incident number (95% CI)	ASR per 100,000	Incident number (95% CI)	ASR per 100,000	
Global	8,283.18 (6,100.92, 9,430.74)	0.365 (0.263, 0.434)	8,700.90 (5,928.97, 10,899.73)	0.320 (0.2249, 0.4135)	−0.3614 (−0.4005, −0.3287)
Male	4,995.25 (3,387.73, 5,995.81)	0.431 (0.287, 0.542)	5,137.79 (3,231.69, 6,939.74)	0.368 (0.222,0.519)	−0.4132 (−0.4572, −0.3788)
Female	3,287.93 (2,318.32, 3,802.94)	0.295 (0.205, 0.359)	3,563.11 (2,293.69, 4,573.74)	0.268 (0.169,0.365)	−0.2636 (−0.3242, −0.2057)
Age					
<5 years	760.38 (367.93, 1,093.07)	0.123 (0.059, 0.176)	539.44 (211.21, 855.50)	0.082 (0.032,0.130)	−1.2968 (−1.3327, −1.2619)
5–9 years	1,564.93 (975.98, 1,995.88)	0.268 (0.167, 0.342)	1,343.99 (800.95, 1,841.40)	0.196 (0.117,0.268)	−1.0384 (−1.1185, −0.9748)
10–14 years	1,959.82 (1,344.94, 2,316.19)	0.366 (0.251, 0.432)	2,392.97 (1,537.98, 2,993.08)	0.359 (0.230, 0.449)	−0.1076 (−0.1731, −0.0546)
15–19	3,998.06 (3,294.19, 4,430.84)	0.770 (0.634, 0.853)	4,424.50 (3,264.80, 5,453.97)	0.709 (0.523, 0.874)	−0.2884 (−0.3228, −0.2483)
SDI					
Low SDI	1,294.44 (664.72, 1,742.90)	0.493 (0.252, 0.706)	2,334.32 (1,189.39, 3,227.65)	0.407 (0.202, 0.585)	−0.4633 (−0.5080, −0.4169)
Low-middle SDI	1,844.14 (1,099.10, 2,391.33)	0.322 (0.186, 0.443)	2,425.11 (1,598.71, 3,338.46)	0.307 (0.197, 0.444)	0.0633 (0.0094, 0.1125)
Middle SDI	1,738.09 (1,117.42, 2,076.66)	0.223 (0.137, 0.275)	1,860.69 (1,178.53, 2,303.01)	0.235 (0.147, 0.301)	0.2828 (0.2434, 0.3173)
High-middle SDI	1,757.37 (1,523.30, 1,915.35)	0.443 (0.378, 0.495)	1,058.43 (911.84, 1,223.97)	0.330 (0.281, 0.385)	−1.0673 (−1.1753, −0.9469)
High SDI	1,640.87 (1,558.67, 1,729.20)	0.589 (0.557, 0.626)	1,016.44 (919.37, 1,121.09)	0.389 (0.350, 0.433)	−1.3860 (−1.4806, −1.2845)

**Table 2:** Age-standardized deaths and average annual percentage change (AAPC) of Hodgkin lymphoma in pediatric and adolescents in 1990 and 2021.

Stratification	1990		2021		AAPC (95% CI)
	Deaths number (95% CI)	ASR per 100,000	Deaths number (95% CI)	ASR per 100,000	
Global	4,311.93 (2,623.38, 5,197.88)	0.190 (0.113, 0.245)	3,385.21 (1,969.38, 4,458.48)	0.125 (0.071, 0.174)	−1.2700 (−1.2995, −1.2441)
Male	2,771.13 (1,520.79, 3,596.28)	0.240 (0.127, 0.327)	2,111.33 (1,144.83, 2,984.40)	0.152 (0.078, 0.227)	−1.3628 (−1.3966, −1.3336)
Female	1,540.81 (839.47, 1,925.70)	0.139 (0.074, 0.185)	1,273.88 (667.40, 1,834.68)	0.096 (0.049, 0.147)	1.1271 (−1.1590, −1.1018)
Age					
<5 years	567.35 (249.49, 851.23)	0.092 (0.040, 0.137)	314.83 (110.60, 510.82)	0.048 (0.017, 0.078)	−2.0956 (−2.1401, −2.0504)
5–9 years	972.87 (519.06, 1,325.05)	0.167 (0.089, 0.227)	607.65 (328.72, 886.41)	0.089 (0.048, 0.129)	−1.9956 (−2.0876, −1.9252)
10–14 years	1,105.33 (650.08, 1,374.07)	0.206 (0.121, 0.257)	996.22 (560.51, 1,332.35)	0.149 (0.084, 0.200)	−1.0442 (−1.1325, −0.9794)
15–19	1,666.38 (1,131.95, 1,938.10)	0.321 (0.218, 0.382)	1,466.50 (931.02, 1,961.07)	0.235 (0.149, 0.314)	−1.0138 (−1.0397, −0.9911)
SDI					
Low SDI	1,093.22 (554.74, 1,476.58)	0.416 (0.211, 0.598)	1,535.08 (767.41, 2,140.75)	0.211 (0.090, 0.343)	−1.2694 (−1.3217, −1.2210)
Low-middle SDI	1,420.39 (842.66, 1,846.22)	0.248 (0.142, 0.346)	1,216.06 (788.62,1,668.20)	0.112 (0.061, 0.169)	−1.2961 (−1.3308, −1.2608)
Middle SDI	1,102.35 (660.14, 1,345.50)	0.141 (0.082, 0.179)	480.35 (302.89, 607.21)	0.046 (0.026, 0.065)	−2.6054 (−2.6384, −2.5707)
High-middle SDI	523.80 (380.99, 610.62)	0.135 (0.095, 0.164)	110.40 (89.74, 139.57)	0.028 (0.022, 0.035)	−4.394 (−4.4742, −4.3164)
High SDI	169.59 (157.01, 180.12)	0.062 (0.057, 0.067)	41.81 (36.28, 47.08)	0.015(0.013, 0.017)	−4.1810 (−4.2455, −4.1111)

**Table 3:** Age-standardized prevalence and average annual percentage change (AAPC) of Hodgkin lymphoma in pediatric and adolescents in 1990 and 2021.

Stratification	1990		2021		AAPC (95% CI)
	Prevalence number (95% CI)	ASR per 100,000	Prevalence number (95% CI)	ASR per 100,000	
Global	43,837.57 (35,229.32,48,251.52)	2.034 (1.623, 2.304)	50,128.16 (36,002.46, 61,732.00)	2.001 (1.441, 2.501)	−0.0748 (−0.1095, −0.0373)
Male	25,301.09 (19,146.13, 29,060.44)	2.278 (1.714, 2.711)	28,847.55 (19,350.10, 38,537.22)	2.210 (1.456, 3.004)	−0.0846 (−0.1409, −0.0375)
Female	18,536.48 (14,466.61, 20,606.72)	1.776 (1.386,2.038)	21,280.61 (14,739.09, 26,523.13)	1.779 (1.225, 2.334)	−0.0873 (−0.1600, −0.0159)
Age					
<5 years	3,390.33 (1,761.79, 4,711.23)	0.547 (0.284, 0.758)	2,631.49 (1,099.78, 4,133.29)	0.400 (0.167, 0.628)	−1.0082 (−1.0419, −0.9754)
5–9 years	7,630.33 (5,216.41, 9,323.89)	1.308 (0.894, 1.598)	7,435.18 (4,720.88, 10,055.97)	1.082 (0.687, 1.464)	−0.6389 (−0.9161, −0.5626)
10–14 years	9,814.68 (7,411.85, 11,185.90)	1.832 (1.384, 2.088)	13,356.97 (9,162.24, 16,541.66)	2.004 (1.374, 2.481)	0.3024 (0.2414, 0.3650)
15–19	23,002.23 (20,258.25, 24,689.44)	4.428 (3.900, 4.753)	16,704.52 (20,852.32, 32,175.16)	4.280 (3.342, 5.156)	−0.1430 (−0.1815, −0.0993)
SDI					
Low SDI	4,456.24 (2,294.32, 6,020.51)	1.717 (0.885, 2.445)	9,675.31 (5,104.42, 13,335.63)	1.784 (0.931, 2.530)	0.1337 (0.0906, 0.1737)
Low-middle SDI	6,817.47 (4,123.14, 8,842.29)	1.208 (0.716, 1.656)	11,606.14 (7,837.45, 16,478.32)	1.568 (1.049, 2.285)	0.9474 (0.9212, 0.9741)
Middle SDI	8,057.33 (5,303.46, 9,590.26)	1.034 (0.653, 1.272)	12,310.88 (7,137.20, 9,521.15)	1.556 (0.989, 1.979)	1.4302 (1.3799, 1.4837)
High-middle SDI	11,761.96 (10,474.70, 12,673.25)	3.140 (2.772, 3.444)	8,251.97 (7,137.20, 9,521.15)	2.860 (2.458, 3.315)	−0.5768 (−0.0683, −0.4575)
High SDI	12,691.67 (12,064.37, 13,362.47)	4.975 (4.707, 5.280)	8,243.81 (7,456.48, 9,081.68)	3.586 (3.231, 3.985)	−1.2288 (−1.3227, −1.1179)

**Table 4:** Age-standardized DALYs and average annual percentage change (AAPC) of Hodgkin lymphoma in pediatric and adolescents in 1990 and 2021.

Stratification	1990		2021		AAPC (95% CI)
	DALYs number (95% CI)	ASR per 100,000	DALYs number (95% CI)	ASR per 100,000	
Global	339,994.82 (205,125.10, 411,551.63)	15.028 (8.814, 19.367)	265,016.48 (153,521.51, 350,078.65)	9.816 (5.540, 13.663)	−1.2890 (−1.3214, −1.2602)
Male	219,686.33 (120,525.08, 286,214.06)	18.994 (10.049, 26.007)	166,503.55 (89,961.88, 235,704.99)	12.049 (6.154, 17.980)	−1.3765 (−1.4115, −1.3450)
Female	120,308.18 (65,110.84, 149,109.02)	10.857 (5.747, 14.454)	98,512.93 (51,709.95, 142,457.72)	7.441 (3.787, 11.373)	−1.1302 (−1.1632, −1.1006)
Age					
<5 years	7.99 (3.52, 11.99)	7.99 (3.52, 11.99)	27,478.62 (9,663.70, 44,575.17)	4.175 (1.468, 6.773)	−2.0962 (−2.1397, −2.0530)
5–9 years	13.92 (7.44, 18.93)	13.92 (7.43, 18.93)	50,888.98 (27,598.36, 74,183.29)	7.407 (4.017, 10.797)	1.9849 (−2.0773, −1.9143)
10–14 years	16.15 (9.52, 20.06)	16.15 (9.52, 20.06)	78,243.51 (44,113.52, 104,498.42)	11.737 (6.617, 15.676)	−1.0351 (−1.1238, −0.9705)
15–19	23.61 (16.07, 28.05)	23.61 (16.07, 28.05)	108,405.37 (39,132.38, 144,551.77)	17.373 (11.079, 23.166)	−1.0008 (−1.0257, −0.9789)
SDI					
Low SDI	86,628.32 (43,688.17, 118,400.07)	32.654 (16.460, 47.338)	120,243.45 (59,587.41, 168,203.94)	20.893 (9.982, 30.329)	−1.3083 (−1.3591, −1.2604)
Low-middle SDI	111,894.74 (66,037.66, 146,639.19)	19.449 (11.045, 27.220)	94,679.55 (61,031.56, 130,559.64)	12.025 (7.457, 17.625)	−1.3310 (−1.3650, −1.2962)
Middle SDI	86,582.68 (51,441.26, 106,116.17)	11.149 (6.389, 14.134)	37,449.75 (23,435.97, 47,463.59)	4.753 (2.928, 6.219)	−2.6280 (−2.6638, −2.5896)
High-middle SDI	41,160.45 (29,797.42, 48,113.35)	10.633 (7.452, 12.977)	8,888.15 (7,195.87, 11,213.95)	2.785 (2.251, 3.514)	−4.3112 (−4.3917, −4.2269)
High SDI	13,526.94 (12,510.41, 14,443.57)	4.922 (4.532, 5.356)	3,637.40 (3,186.01, 4,140.16)	1.404 (1.216, 1.623)	−3.9104 (−4.2160, −4.0697)

### HL burden stratified by sex and age in children and adolescents in 2021

In total, compared with women, men had a higher HL burden (Tables 1–4). The global prevalence rate in men (ASPR = 2.210) was 24.2% higher than that in women (ASPR = 1.779) in 2021. The incidence rate in men (ASIR = 0.368) was slightly 37.3% higher than that in women (ASIR = 0.268). The mortality rate in men (ASMR = 0.152) was 58.3% higher than that in women (ASMR = 0.096). The DALY rate in men (ASDR = 12.049) was 61.9% higher than that in women (ASDR = 7.441).

Consistent with previous findings, the peak values of ASPR, ASMR, ASIR, and ASDR were all observed in the 15–19year age group, showing a progressively increasing trend across the 0–4, 5–9, 10–14 and 15–19 years age groups. The ASPR, ASMR, ASIR, and ASDR values in the 15–19 years age group were 4.280, 0.235, 0.709, and 17.373, respectively.

In 2021, the burden of HL among children and adolescents demonstrated a pronounced socioeconomic gradient, with ASIR ranging from 1.78 per 100,000 in low-SDI regions to 3.59 per 100,000 in high-SDI regions. Conversely, the ASDR was substantially higher in low-SDI regions (20.89 per 100,000) compared to high-SDI regions (1.40 per 100,000), reflecting a nearly 15-fold disparity in health loss despite lower incidence.

### HL burden stratified by SDI in children and adolescents in 2021

In 2021, the burden of HL among children and adolescents demonstrated a pronounced socioeconomic gradient, with ASIR ranging from 1.78 per 100,000 in low-SDI regions to 3.59 per 100,000 in high-SDI regions. Conversely, the ASDR was substantially higher in low-SDI regions (20.89 per 100,000) compared to high-SDI regions (1.40 per 100,000), reflecting a nearly 15-fold disparity in health loss despite lower incidence.

### Trends in HL burden among children and adolescents from 1990 to 2021

As Table 1 showed, HL epidemiology among children and adolescents exhibits diverging temporal signatures between crude caseloads and standardized rates. Global HL cases among children and adolescents increased 5.04% (8,283 in 1990; 8,700 in 2021), while ASIR declined from 0.365/100,000 in 1990 to 0.320/100,000 in 2021, with the AAPC of −0.36%, underscoring demographic expansion's role in offsetting rate reductions. However, a significant decline was observed globally in the number of deaths, ASMR, DALYs and ASDR of HL among children and adolescents from 1990 to 2021. In 2021, Compared with those in 1990, the number of deaths and ASMR decreased by 21.5% (3,385 vs. 4,312) and 34.2% (0.190 vs. 0.125 per 100,000) in 2021, respectively. The DALYs and ASDR declined by 22.1% (339,995 vs. 265,016) and 34.7% (15.028 vs. 9.816 per 10,0000), respectively.

**Figure 1:**
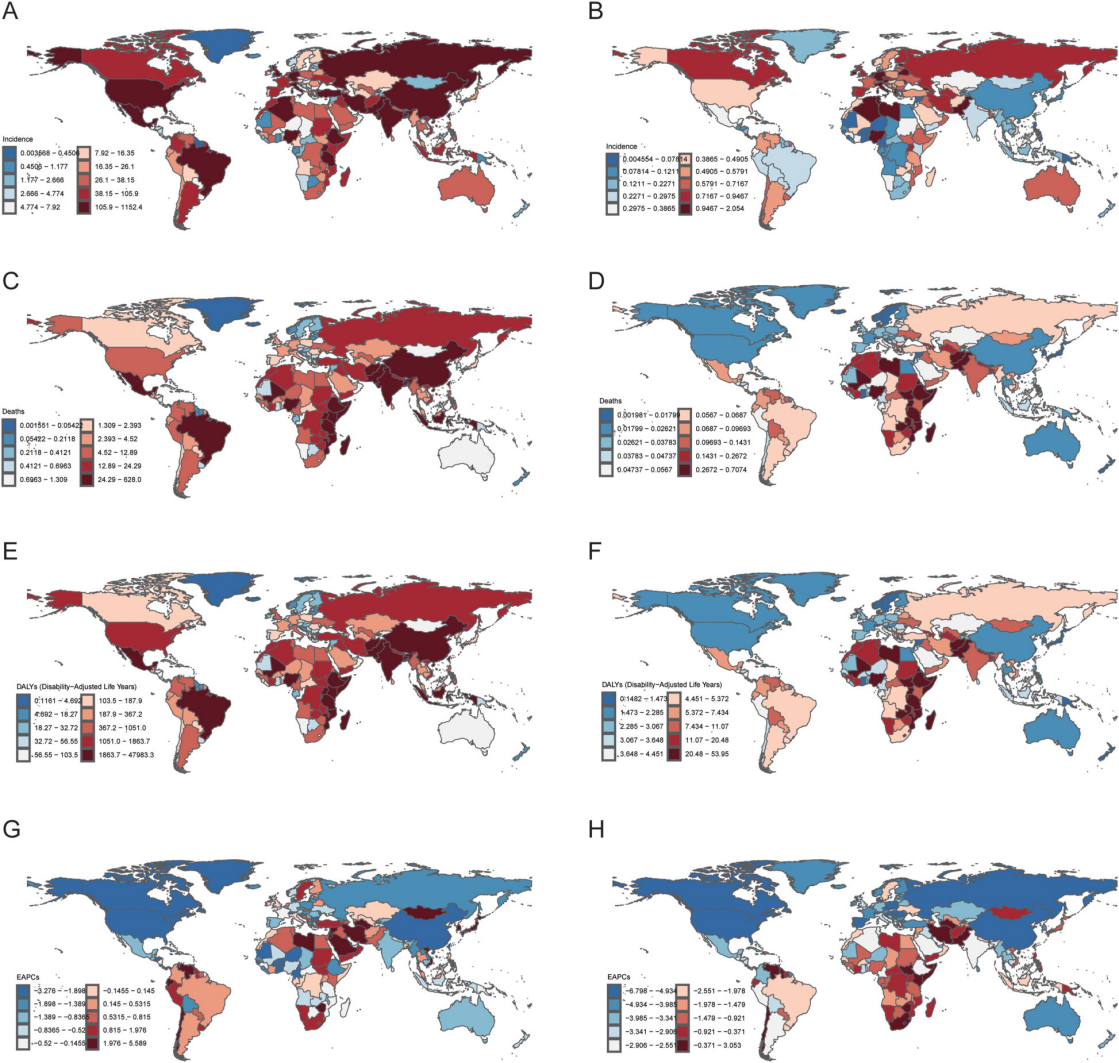
National burden of Hodgkin lymphoma (HL) in people aged < 20 years in 2021. **(A)** Incidence number. **(B)** Age-standardized incidence rates (ASIR). **(C)** Mortality number. **(D)** Age-standardized mortality rates (ASMR). **(E)** Disability-adjusted life years (DALYs) number. **(F)** Age-standardized DALYs rates (ASDR). **(G)** Estimated annual percentage change (EAPC) number. **(H)** Estimated annual percentage change (EAPC) rate. The source code of ggmap is available at: https://github.com/dkahle/ggmap

The HL burden among children and adolescents exhibited significant disparities across SDI regions. High-SDI areas show substantial declines in incidence (AAPC: −1.39%) and DALYs (AAPC: −3.91%), reflecting advanced healthcare. In contrast, low- and middle-SDI regions face increasing incidence (AAPC: 0.06% and 0.28%) and slower mortality improvements, with the highest DALY rates (20.89) persisting. In 2021, 15 of 204 countries and regions reported more than 100 cases of children and adolescents HL, which accounted for 64.9% of the global HL cases. Of noted was a concentration in low to middle socio-demographic Index (SDI) areas. There were 110 countries and regions where the ASIR exceeded the global average, with the top five being the Principality of Monaco, the Republic of San Marino, the Hellenic Republic, the Republic of Malta, and the State of Libya. The estimated annual percentage change (EAPC) values revealed that among the 204 countries and regions, 95 exhibited an upward trend in HL incidence, with 37 of these having an EAPC exceeding 1.0 and 5 surpassing an EAPC of 3.0. Notably, the majority of these countries are located in upper-middle to high SDI regions, characterized by advanced economies and medical infrastructures. Among the countries with the fastest-growing HL incidence, Saint Kitts and Nevis (with a high-middle SDI) led significantly with an EAPC of 6.80 (95% CI: 4.35, 9.32), followed by Chile, Iran (Islamic Republic of), and Singapore. In contrast, the United States of America spearheaded the decline in HL incidence, with an EAPC of −3.28 (95% CI: −3.63 to −2.92) (Figure 1).

### SDI-stratified temporal dynamics

**Figure 2:**
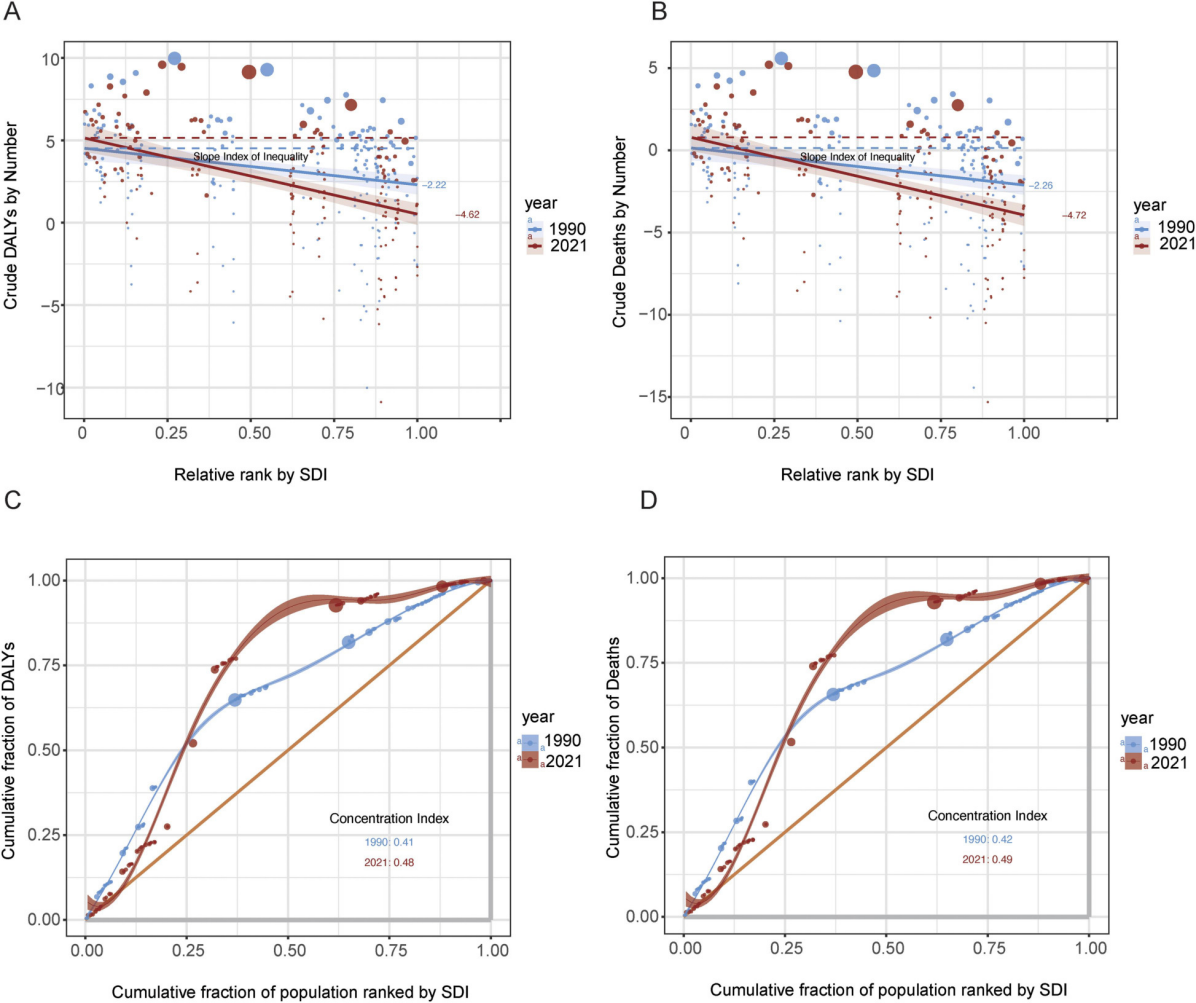
Inequality analysis of DALYs and deaths by SDI, 1990–2021. **(A)** Slope Index of Inequality (SII) for crude DALYs across SDI-ranked populations in 1990 and 2021. **(B)** SII for crude deaths using the same SDI ranking. **(C)** Concentration curves and concentration indices for DALYs by SDI rank in both years. **(D)** Concentration curves and concentration indices for deaths by SDI rank in both years.

The quantification of health inequalities reveals significant and persistently widening global disparities in HL burden among children and adolescents. Between 1990 and 2021, the Slope Index of Inequality (SII) for DALYs deteriorated markedly from −2.22 to −4.62 per 100,000 population (*Δ* =  + 108%, *P* = 0.002), indicating that low-income countries bore 4.62 excess annual DALYs per 100,000 population compared to high-income countries. This absolute disparity was paralleled by a worsening Concentration Index (CI) from 0.41 to 0.48, reflecting an increasing concentration of the HL burden in low- SDI regions. Similar inequality patterns were observed in mortality burden, with the SII declining from −2.26 to −4.72 per 100,000 and the CI rising from 0.42 to 0.49 (Figure 2).

**Figure 3:**
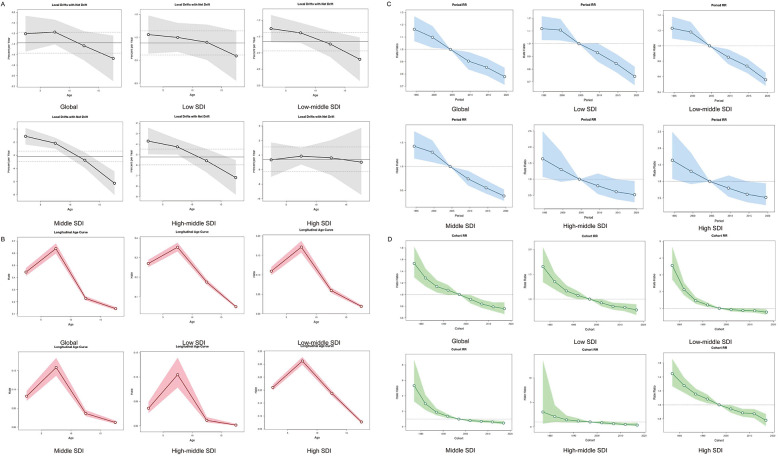
Age, period and birth cohort effects on HL mortality in children and adolescents by APC models. **(A)** Local drift of mortality from 1992 to 2021 for HL in children and adolescents for four age groups. The dots and shaded areas denote the local drift (i.e., annual percentage change of age-specific incidence, % per year) and their corresponding 95% CIs. **(B)** The age effect is depicted through the longitudinal rates specific to age, which are adjusted for variations across different birth cohorts, taking into account the period-specific deviations. **(C)** Period effects are shown through the relative risk of HL mortality during different periods, calculated as the ratio of the age-specific rates from the period from 1990–1994 to 2015–2019, with the baseline period set as 2000–2004. **(D)** Birth cohort effects are demonstrated by the cohort relative risk of mortality and calculated as the ratio of age-specific rates from 1980 to 1990 cohort to 2010–2020 cohort, with the reference cohort set at 1990–2000. The dots and shaded areas denote the mortality rates or rate ratios and their corresponding 95% CIs. HL, Hodgkin lymphoma; SDI, social demographic index.

Age-period-cohort modeling indicated a synchronized global decline in mortality (net drift = −1.38%/year, 95% CI: −1.58 to −1.18), equivalent to a 28.4% cumulative reduction over three decades. Declines were most pronounced among adolescent aged 15–19 years globally (local drift = −1.673) and in medium-high–SDI regions (local drift = −7.184). Age effects were consistent worldwide, with peak HL risk occurring between ages 5–10, followed by a decline through adolescence. Period risks were elevated in the 1990s relative to the 2002–2006 reference period, and cohort analysis revealed a continuous risk reduction in successive birth cohorts after 1995 (Figure 3).

### Prediction of HL disease burden in children and adolescents population before 2040

**Figure 4:**
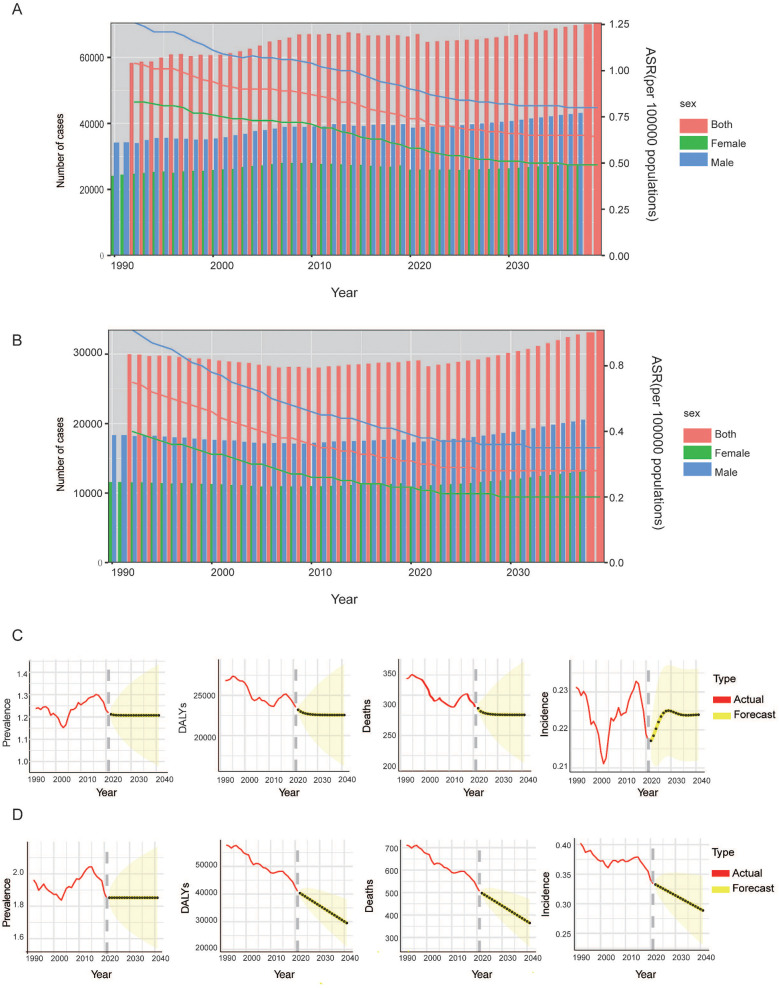
Projects the ASIR **(A)**, ASMR **(B)** and numbers of HL in all population for global from 2022 to 2040. Projects the ASIR, ASPR, deaths and DALYs number of HL in female **(C)** and male **(D)** children and adolescents for global from 2022 to 2040. ASIR, age-standardized incidence rate; ASMR, age-standardized mortality rate; ASPR, age-standardized prevalence rate; DALYs, disability adjusted life years.

The Nordpred model is used to predict the future trend of incidence number of HL in the global population (Figure 4A) and the number of deaths (Figure 4B) caused by HL from 2022 to 2040. The ARIMA model was used to predict HL burden among children and adolescents populations of different genders around the world (Figures 4C and 4D). By 2040, it is expected that the incidence, mortality and DALYs of male children and adolescents will continue to decline, and the disease burden will gradually decrease. In terms of prevalence, it is expected that the global male children and adolescents population will remain flat by 2040, with no obvious downward or upward trend, and the disease prevalence burden will not change significantly. On the other hand, the global prevalence and mortality of female children and adolescents are expected to increase in 2040. It is expected that the ASIR will rise to 0.21 (95%CI: 0.23, 0.24) in 2030, and then decline slightly. By 2040, the ASIR will reach 0.22(95%CI: 0.21, 0.24). At the same time, by 2040, the number of deaths from HL in female children and adolescents will continue to rise. Males and females are expected to have similar trends of remaining flat in the prevalence and continuing to decline in the DALYs by 2040. It is estimated the prevalence rate of HL will be 1.85 (95%CI: 1.55, 2.16) and 1.21(95%CI:0.99, 1.43), and the DALYs will be 30103 (95%CI: 21712, 38,495) and 22,559 (95%CI: 16,431, 28,686) in males and females, respectively.

## Discussion

This study delineated the global burden of HL among children and adolescents from 1990 to 2021 by leveraging the GBD 2021 dataset. The key findings revealed an overall decline in ASIR and a more substantial reduction in DALYs, yet these trends are characterized by significant disparities across sex, age, and SDI regions. The general decline in ASIR, coupled with a sharper decrease in DALYs, is indicative of considerable progress in therapeutic efficacy and patient survival over the past three decades. This pattern is consistent with advances in multimodal therapy and supportive care documented in high-income settings (14).

A notable and persistent sex disparity was observed, with males demonstrating higher ASIR and DALY rates than females in both 1990 and 2021. This is consistent with previous research by Pan Tao et al., who analyzed the global burden of hematologic diseases and found that the incidence, mortality, and DALYs rates of Hodgkin lymphoma were higher in males than in females worldwide. Notably, among all populations under 20 years of age, the disease burden was consistently greater in males, with the most pronounced sex-based disparity observed in the 5–9 years age group (15). The somewhat slower AAPC rate of decline in male incidence warrants further investigation into sex-specific disparities in healthcare access or the evolution of risk factors.

Age-specific analysis revealed the most pronounced improvement in the under 5 age group, likely attributable to distinct early-life immune ontogeny and lower exposure to established risk factors such as EBV. A prior study demonstrated that EBV-positive incidence in males peaks across three distinct age groups (5–15, 25–40, and 55–70 years), compared to only two (25–40, 55–70 years) in females, offers a plausible explanation for the observed sex disparity, potentially implicating a broader age-specific susceptibility to EBV-associated oncogenesis in males (16). Despite a favorable temporal trend, underscores this period as high-risk and highlights the necessity for sustained vigilance and targeted interventions in this demographic.

A critical finding is the divergent epidemiological transition across SDI regions. High-SDI regions demonstrated significant declines in both incidence and burden, consistent with robust healthcare systems and effective control of associated infections. In contrast, rising ASIRs in many low- and middle-SDI regions illustrate a shifting burden towards economically developing areas, a pattern similarly observed for other infection-related cancers. The high prevalence in high-SDI regions and the high incidence in low-SDI regions align with previous findings (17), which support the “delayed infection” hypothesis (18–20). This model suggests that adolescent from more affluent conditions may have a higher HL risk due to a lack of early microbial exposure, whereas children from poorer conditions may experience a higher incidence attributed to early-life exposures. The attenuated decline in DALYs in these settings underscores the dual challenge of rising incidence and barriers to accessing curative treatments, emphasizing an urgent need for equitable health system strengthening.

The age-period-cohort analysis identified a consistent peak in age-specific risk around the 5–9 years group across all SDI regions. The favorable period and cohort effects globally likely stem from secular improvements in nutrition, sanitation, and management of immunocompromising conditions like HIV, which have collectively reduced population-level risk. The global decline in HL burden is consistent with improved control of known risk factors, such as the expansion of antiretroviral therapy leading to improved immune function in people living with HIV. These observations suggest that continued public health gains in these areas are crucial for sustained progress.

Although the ASIR of HL in children and adolescents decreased during 1990–2021, the absolute number of incident cases continued to rise. This apparent discrepancy can be partly explained by population growth. According to the United Nations World Population Prospects, the global population of children and adolescents (aged 0–19) increased from 25.2 billion in 1990 to 29.3 billion in 2021 (21). Notably, low-income regions, particularly Africa, exhibited both the highest proportion and most rapid growth of young populations. By coincidence, higher HL incidence was observed in these regions. Furthermore, advancements in medical imaging and histopathological techniques, along with improved training of pathologists, have enhanced diagnostic sensitivity. Additionally, evolving diagnostic criteria—such as the widespread incorporation of EBV testing—have refined HL subclassification and may indirectly influence incidence reporting patterns over time.

Future strategies must be tailored to specific demographic and socioeconomic contexts. Priorities include reinforcing primary prevention (e.g., hygiene to reduce EBV transmission, vaccination programs) in regions with rising incidence, and strengthening diagnostic and treatment capacity in low-resource settings to ensure equitable survival gains. Global cancer control strategies require recalibration to prioritize HL management in young populations residing in resource-limited settings. Actionable measures should include strengthening histopathological infrastructure and ensuring reliable access to essential chemotherapeutic agents, enhancing early detection through the integration of HL recognition into primary healthcare training and community health awareness initiatives to mitigate diagnostic delays, and establishing international collaborations for capacity building, knowledge transfer, and technology dissemination. Moreover, intervention strategies must be adapted to local resource constraints—via the implementation of simplified diagnostic and treatment protocols and task-sharing care models—to ensure feasibility and sustainability. Global cancer control strategies must be recalibrated to ensure that children and adolescents HL is no longer a neglected priority in low-resource settings.

The current study had several limitations. In many low-SDI countries, constrained diagnostic capacity and underdeveloped cancer registries likely lead to under-ascertainment of HL cases (22). The reliance on GBD modeling in the absence of primary data for some regions introduces uncertainty in estimating age, period, and cohort effects. Furthermore, significant heterogeneity in diagnostic criteria and pathology expertise across settings may affect the comparability of HL subtype distributions and EBV status classification. The retrospective nature of the GBD data may also impact the timeliness of findings. Finally, despite statistical adjustments, residual confounding may persist due to sparse data and unmeasured factors in certain populations (23). These factors highlight the need for cautious interpretation of cross-country comparisons and reinforce the urgency of improving cancer surveillance infrastructure worldwide.

In conclusion, the burden of HL among children and adolescents is increasing. A significant difference by sex, age and regions was observed. Although a global decline in ASIR is projected by 2040, absolute case numbers will continue to rise in low-SDI countries. Thus, a differentiated strategy of disease control and prevention is needed in the future.

## Data Availability

The original contributions presented in the study are included in the article/Supplementary Material, further inquiries can be directed to the corresponding authors.
